# The role of the hippocampus and retrosplenial cortex in spatial memory: a double blind anodal transcranial direct current stimulation study

**DOI:** 10.3389/fnhum.2025.1661310

**Published:** 2025-10-20

**Authors:** Rosalia De Biase, Sara Esposito, Emma Chiaramello, Marta Parazzini, Laura Sagliano

**Affiliations:** ^1^Department of Psychology, University of Campania Luigi Vanvitelli, Caserta, Italy; ^2^National Research Council (CNR), Institute of Electronics, Computer and Telecommunication Engineering (IEIIT), Milan, Italy

**Keywords:** spatial memory, retrosplenial cortex, hippocampus, tDCS, perspective shift, spatial updating

## Abstract

**Introduction:**

Spatial memory supports orientation and navigation by integrating multiple spatial reference frames. Neuroimaging and lesion studies implicate the hippocampus (HIP) and retrosplenial cortex (RSC), but causal evidence from non-invasive brain stimulation is limited.

**Methods:**

Eighteen participants performed a spatial localization task in a virtual room under three stimulation conditions: anodal transcranial direct current stimulation (tDCS) over the left RSC, anodal tDCS over the left HIP, and sham. Task conditions varied in reference frame (viewer-, object-, room-centered) and perspective shift (0°, 45°, 135°). Accuracy was analyzed with non-parametric statistics.

**Results:**

Performance declined with increasing viewpoint rotation, especially in room-centered trials. RSC stimulation selectively reduced accuracy in room-centered trials with large perspective shifts (135°), whereas HIP stimulation did not significantly modulate performance.

**Discussion:**

Findings provide causal evidence for the involvement of the RSC in viewpoint-invariant spatial updating, supporting its role in integrating stable environmental cues. HIP stimulation yielded no reliable behavioral effects, suggesting functional specificity of the RSC and highlighting the challenges of modulating deep cortical structures with tDCS.

## 1 Introduction

Spatial memory, the ability to remember the locations of objects in the environment, is a core cognitive function supporting orientation, navigation, and goal-directed behavior (Marchette et al., 2011). This ability is based on the integrating multiple spatial reference frames, particularly egocentric (observer-centered) and allocentric (environment-centered) representations. Egocentric representations encode spatial relations relative to the body and viewpoint of the observer, enabling real-time interaction with the surrounding space (Simons and Wang, 1998; Wang, 2012). In contrast, allocentric representations are viewpoint-independent and capture the spatial configuration among objects or environmental features, forming the basis of cognitive maps (O’Keefe, 1990; Wang, 2012). The flexible transformation of information between these two systems is essential for spatial updating and mental navigation across changing perspectives (Burgess et al., 2006; Byrne et al., 2007).

Previous neuroimaging studies showed that egocentric and allocentric reference frames engage different neural networks (Committeri et al., 2004; Galati et al., 2000; Zaehle et al., 2007) with the egocentric frame involving posterior parietal and premotor areas that represent spatial relations relative to the observer’s body, and the allocentric frame involving medial temporal lobe structures, such as the hippocampus (HIP) and parahippocampal cortex, which support viewpoint-independent representations of spatial relationships. Moreover, two qualitatively different types of allocentric coding can be dissociated: one based on the spatial relationships among arbitrary movable objects (object-based reference frames), and another based on fixed features of the environment, such as environmental or landmark-based reference frames (Galati et al., 2010; Sulpizio et al., 2013). According to Galati et al. (2010), these reference frames are differentially related to specific brain regions: the medial parietal and occipito-temporal cortices are activated during the use of stable environmental landmarks, supporting the encoding of allocentric environment-based information, whereas anterior parahippocampal and retrosplenial areas are more strongly recruited when participants rely on landmark-based reference. Sulpizio et al. (2013) further demonstrated that the retrosplenial cortex (RSC) and parahippocampal gyrus are selectively involved in processing allocentric spatial representations anchored to stable elements of the environment, and that the RSC is uniquely modulated by changes in the observer’s viewpoint, thus playing a key role in spatial updating and coordinate transformations between reference frames. Among these regions, the HIP and the RSC have been consistently highlighted as core nodes in the neural network underlying spatial memory and navigation (Vann et al., 2009; Sulpizio et al., 2013; Burgess et al., 2002).

The HIP is crucially involved in the encoding of allocentric spatial relations and episodic context. It is also responsible for the construction of internal maps that allow navigation in both real and imagined environments (Burgess et al., 2002; Maguire et al., 1998; Bird and Burgess, 2008). Notably, a functional lateralization has been consistently reported: while the right HIP supports accurate spatial mapping and navigation (Bohbot et al., 1998; Burgess et al., 2002), the left HIP appears more involved in episodic and verbal contextual memory, particularly in reconstructing spatial scenes from sequential or incomplete visual cues (Spiers et al., 2001; Maguire and Frith, 2003). This dissociation is supported by lesion studies showing that damage to the right HIP impairs spatial memory and navigation, whereas left-sided lesions are more associated with deficits in verbal recall (Bohbot et al., 1998).

The RSC, in turn, represents a functional interface between egocentric representations encoded by the parietal cortex and allocentric maps reconstructed within the HIP (Byrne et al., 2007; Vann et al., 2009; Mao et al., 2017; Chen et al., 2024). Indeed, recent evidence has further characterized the RSC as a multifunctional hub, integrating allocentric and egocentric spatial information through its strong reciprocal connectivity with the HIP and other medial temporal and parietal areas. Alexander et al. (2023), proposed a functional model in which the RSC acts as a dynamic interface for transforming spatial representations across perspectives and time, predicting its involvement in both perceptual and mnemonic domains. RSC receives and sends reciprocal projections to medial temporal and posterior parietal areas (Ekstrom et al., 2014) allowing the transformations of spatial information and supporting the orientation within complex environments. RSC is crucial for creating unified spatial representations by integrating perspectives across time during perception and imagination (Miller et al., 2014; Alexander and Nitz, 2017; Chen et al., 2024). RSC is sensitive to scene permanence and landmark stability, even for unfamiliar environments, and responds preferentially to viewpoint changes anchored to stable cues (Sulpizio et al., 2013; Auger et al., 2015) with specific activation peaks localized in the left hemisphere (Sulpizio et al., 2013). Indeed, evidence suggests that the left retrosplenial cortex is particularly sensitive to scene coherence, landmark permanence, and the transformation of spatial representations across shifting viewpoints (Alexander and Nitz, 2017; Claessen and van der Ham, 2017; Ruggiero et al., 2014). de Landeta et al. (2020) demonstrated that RSC activity is not only linked to spatial navigation but also to broader contextual memory processes, supporting the flexible retrieval of scene-based information. The left medial RSC shows a selective involvement in episodic memory and scene imagery (Chrastil et al., 2018). Previous neuroimaging studies showed its activity increases with the cognitive load of updating spatial information across large perspective shifts and when integrating cues from different viewpoints (Sulpizio et al., 2013). The RSC also encodes object positions relative to stable environments (Galati et al., 2010) and supports “offline” spatial updating for reorientation and scene recognition (Galati et al., 2010; Sulpizio et al., 2013). Further evidence come from the lesion studies demonstrating that right hippocampal damage impairs allocentric navigation (Maguire et al., 1998; Bohbot et al., 1998; Burgess et al., 2002) and that retrosplenial damage may determine topographical disorientation, episodic memory deficits, and impairments in spatial reorientation (Song et al., 2020; Valenstein et al., 1987). Moreover, Maeshima et al. (2001) showed that a left retrosplenial lesion produce marked impairments in both verbal and visual memory, along with spatial disorientation. This case highlights the integrative role of the left RSC in supporting memory and orientation processes, especially through its connections with the HIP, anterior thalamus, and visual areas. By contrast, Cammalleri et al. (1996) described a case of transient topographical amnesia associated with a focal lesion in the right retrosplenial and posterior cingulate cortices, further supporting the critical role of these medial parietal structures and of the right hemisphere, in spatial memory processing and navigation.

Despite substantial evidence from neuroimaging and lesion studies, causal evidence on the distinct contributions of the HIP and RSC to spatial memory is still lacking. This gap is largely due to anatomical constraints: both regions are located deep within the brain, which makes them inaccessible to direct non-invasive brain stimulation (NIBS) approaches. Conventional tDCS montages do not allow for focal stimulation of such structures, as the induced electric field decays rapidly with depth and inevitably spreads across overlying cortical areas (Bjekić et al., 2021). For this reason, most prior studies have refrained from attempting direct stimulation of the HIP or RSC, as the lack of focality would compromise the interpretability of the effects. Instead, an effective strategy, as suggested by network-based models (Kim et al., 2016), is to target superficial cortical ‘access points’ that are strongly connected to these regions, thereby influencing their activity indirectly via established cortico-hippocampal and cortico-retrosplenial pathways.

Indeed, clinical studies have also shown that anodal transcranial direct current stimulation (tDCS) over the right temporo-parietal junction can improve hippocampal-dependent spatial learning, as in Philippen et al. (2024), likely via modulation of the parietal–hippocampal network. Other studies (e.g., Živanović et al., 2021) focused on superficial regions, for example the parietal cortex, to modulate and enhance spatial working memory. These findings demonstrated that tDCS is a promising tool for probing the causal role of brain regions in spatial memory.

The current study aimed to investigate the role of RSC and HIP in spatial memory by applying anodal tDCS over the left RSC and left HIP in a double-blind within-subject design. We conducted a dedicated montage simulation study to maximize the electric field in cortical regions anatomically adjacent to, and functionally connected with, the left RSC and HIP, while minimizing spread to non-target areas. Participants performed a spatial memory task in which they encoded the position of a target object within a virtual room, using either themselves (viewer-centered frame) a set of stable, familiar environmental features (room-centered frame) or a configuration of arbitrary, movable objects (object-centered frame) as reference. After the initial encoding, the same environment was presented from a different viewpoint, and participants were asked to determine whether the target object remained in the same spatial location. Crucially, the definition of “same” location adhered to the reference frame used during encoding, either relative to the room layout or to the object array. Based on previous literature, we expected that the online tDCS stimulation on RSC and the HIP might differentially affect performance depending on the spatial reference frame and the extent of viewpoint change. The RSC has been consistently implicated in spatial updating and in transforming egocentric and allocentric information, particularly when stable environmental features are used to maintain orientation across changes in perspective (Byrne et al., 2007; Sulpizio et al., 2013; Alexander and Nitz, 2017; Chen et al., 2024). In contrast, the HIP is thought to support the encoding and retrieval of allocentric spatial representations and the construction of relational scene memory (Burgess et al., 2002; Bird and Burgess, 2008). Accordingly, we hypothesized that RSC stimulation might have a stronger impact when the task involves viewpoint shifts under room-based conditions, whereas hippocampal stimulation might modulate performance more generally across frames, reflecting its broader role in relational memory construction and retrieval.

## 2 Methods

### 2.1 Participants and experimental design

This study utilized a within-subject, double-blind, online tDCS design. The experiment followed a 3 × 3 × 3 fully within-subjects factorial design, with factors including: stimulation site (left RSC, left HIP, or sham tDCS), spatial reference frame (viewer-, object-, and room-centered), and perspective shift (0°, 45°, and 135°). *A priori* power analysis conducted with G*Power 3 with a medium effect size (Cohen’ s f) of 0.25 (Cohen, 2013), an alpha of 0.05 and the power set to 0.95 based on GPower 3 default setting demonstrated that the minimum total sample size is 11 participants to conduct repeated measures ANOVA with three within-subject factors: stimulation (RSC, HIP, sham), spatial reference frame (viewer, object, and room), and perspective shift (0°, 45° and 135°). However, to ensure a fully counterbalanced and orthogonal design, we employed a Latin square procedure to control for order effects across the three stimulation conditions, resulting in a final sample size of 18 participants. Thus, 18 healthy participants were recruited from the student population of the University of Campania “Luigi Vanvitelli.” They were aged between 19 and 25 years and comprised nine males (*M* = 22.2 years; SD = 2.16 years) and nine females (*M* = 23.5 years; SD = 1.5 years). Participants were naïve to the study aims and predictions and gave their written informed consent to participate after having received a complete description of the study procedures. Eligibility for tDCS stimulation was assessed through a semi-structured interview. We planned to exclude individuals who declared a history of epileptic seizures or convulsions, fainting episodes, head injuries with loss of consciousness, hearing disorders, pregnancy, presence of pacemakers or metallic implants, use of medication, or previous spinal surgery. However, no participant met exclusion criteria. Participants did not receive any monetary compensation or other incentives for their participation. The studies involving humans were approved by Institutional ethics committee of the Department of Psychology, University of Campania “Luigi Vanvitelli” (n. 25/2024). The studies were conducted in accordance with the local legislation and institutional requirements. The participants provided their written informed consent to participate in this study.

### 2.2 Stimuli

**Figure 1:**
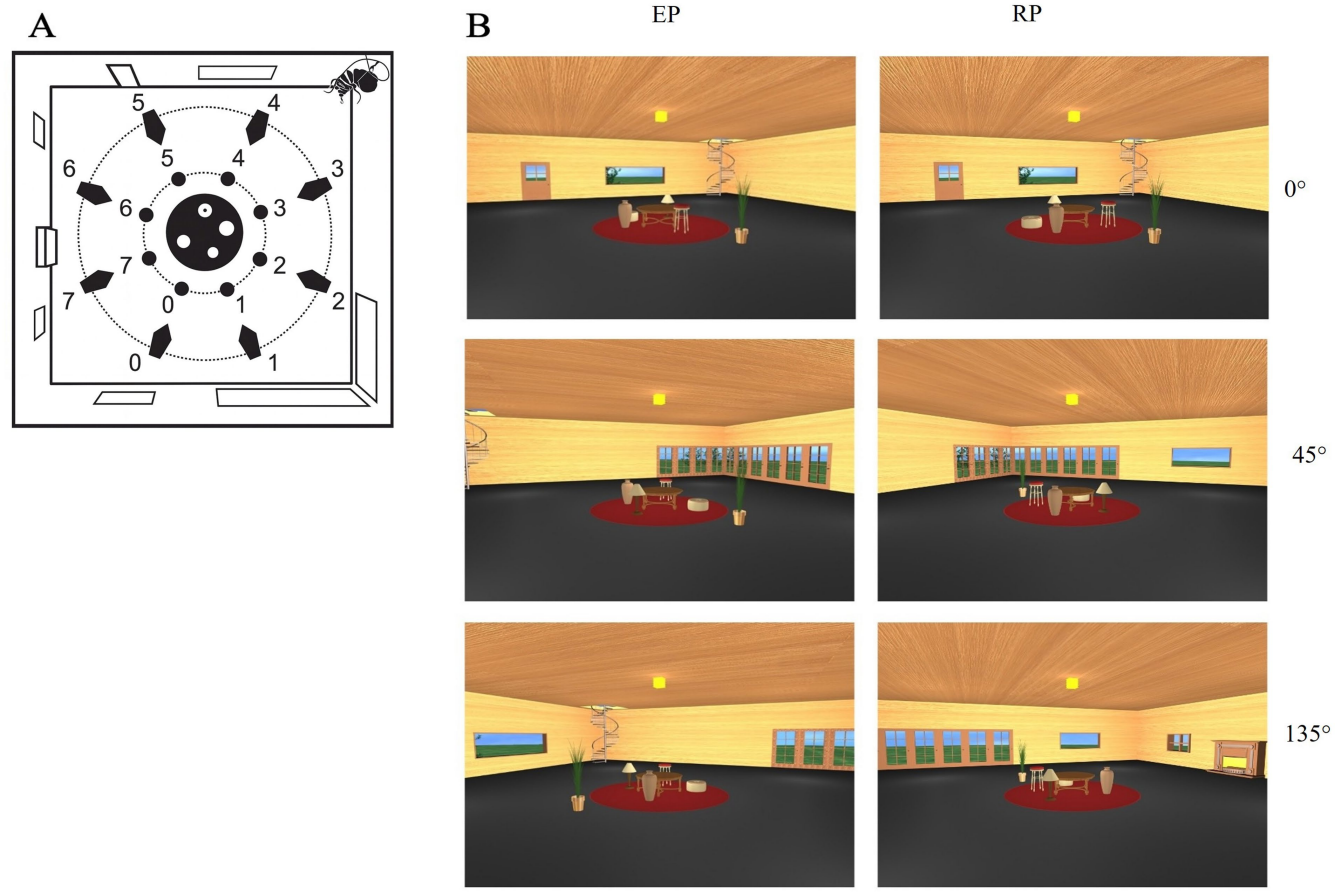
**(A)** View of the virtual environment (Sulpizio et al., 2013) used in the experiment. Blue numbers indicate the possible positions of the virtual cameras used to generate the snapshots employed as stimuli. Green numbers indicate the possible positions of the target object (plant), arranged along a green circular trajectory around the central furniture layout (in red). **(B)** Examples of encoding and recognition views at 0°, 45°, and 135° angular disparity in the room condition. Each row shows a pair of encoding (left) and recognition (right) perspectives corresponding to the same trial.

The stimuli used in this study were developed by Sulpizio et al. (2013) and consisted of a photorealistic virtual environment depicting a living room. The room featured a square floor plan with four enclosing walls and was furnished with both stable and moving objects. The stable landmarks, affixed to the walls, included a door, two small grating windows, two large windows, a spiral staircase, a fireplace, and a large corner French window. These elements served as room-based cues and remained invariant across trials. At the center of the room, five objects changing position (a round table, a stool, an ottoman, a vase, and a lamp) were placed on a circular carpet. These objects lacked intrinsic orientation, meaning that their spatial processing was viewpoint-invariant and did not facilitate performance through directional cues. A target object, a plant, was positioned on the floor close to the carpet and could appear in different locations across trials. The stimuli consisted of static snapshots of the room taken from eight distinct viewpoints distributed at 45° intervals around the center of the room. For the present study, we only used three perspective change 0°, 45°, 135°; Figure 1, panel A. Each snapshot depicted a specific perspective and included both the arbitrary objects and a subset of the stable wall-based cues, depending on the viewing angle. The target object also varied in position, occupying one of several predefined locations along an inner imaginary circle centered within the room. This design allowed for the manipulation of both viewing perspective and spatial reference frame, enabling the systematic assessment of spatial memory performance across egocentric and allocentric conditions. A full description of the stimuli and their generation procedures is available in Sulpizio et al. (2013).

### 2.3 Experimental paradigm

The memory task required participants to determine if a target (plant) object’s position changed between study and test images, evaluated with respect to one of three specified reference frames: the observer’s viewpoint, stable room objects, or arbitrary objects on a carpet. The task was structured into three distinct blocks, each requiring the encoding and recognizing the target object’s location based on a specific spatial reference frame (viewer-centered, room-centered, or objects-centered). The specific block was specified at the beginning of each block. Within each block, ‘shifting trials’ (50% of total) involved a repositioning of the plant relative to the current reference frame, alongside a viewpoint shift of either 45° or 135°. The remaining trials featured no viewpoint shift (0°) relative to the reference frame (‘no-shift trials’). Trial presentation was pseudo-randomized within each session to avoid order effects. In each trial, during the encoding phase, participants viewed a static image of the virtual room from a specific perspective for 4 s and were instructed to encode the location of the target object (the plant) relative to the indicated reference frame. Following a 2-s interval, the recognition phase started with a second image, presented for 10 s, that could vary from the encoding image in terms of the rotation of the room, the set of objects, and the plant itself (Figure 1, panel B). These elements could be rotated independently. Each combination of reference frame (*n* = 3) and viewpoint change (*n* = 3) was repeated across 20 trials, yielding a total of 180 trials per session. For each trial, Responses were made via keyboard. Accuracy was recorded for each trial.

In the viewer-centered block, participants were instructed to rely on the position of the plant relative to the participant’s viewpoint to determine whether the plant occupied the same or a different spatial position (egocentric reference frame). In the room-centered block, participants were instructed to rely on the structure and stable cues of the room to determine whether the plant occupied the same spatial location relative to the stable room features, requiring a mental rotation of their own perspective to match the new viewpoint (allocentric-room reference frame). In the objects-centered block, participants were instructed to rely on the moving objects on the carpet to determine whether the plant occupied the same spatial location relative to them (allocentric-objects reference frame). In the “different perspective” conditions (45° or 135°), the viewpoint, the set of objects, and the room were always rotated relative to one another, allowing the clear dissociation of egocentric, allocentric-room, and allocentric-object reference frames.

### 2.4 Transcranial direct current stimulation

Stimulation has been delivered by a constant current stimulator (BrainStim) using a pair of surface saline-soaked sponge electrodes. Stimulation parameters have been determined by means of a computational model (for details see Supplementary material) of the electric field amplitude distributions. Simulations were conducted using the simulation platform Sim4Life (ZMT Zurich MedTech AG) and the highly detailed anatomical model of the human head named “MIDA” (Iacono et al., 2015) following an approach already used in literature (see, e.g., Sagliano et al., 2019), to identify electrodes positioning able to induce the highest and most widespread electric field amplitude distribution over the RSC and hippocampal region, thus obtaining effective stimulation of the two areas of interest.

Differences in skull dimension and form are dealt with calculating several measures over the participants’ scalp (e.g., skull circumference, nasion-inion distance) so that the target sites could be selected to comply with the International 10–20 Electrode Placement System (Klem et al., 1999). For the RSC stimulation, we placed a 2.5 × 2.5 cm anodal electrode centred over the hypothetical RSC projection on the skin, identified by the model between T3 and T5 while the 2.5 × 2.5 cm cathodal electrode was placed over O1. For the HIP stimulation, the same 2.5 × 2.5 cm anodal electrode was placed over the hypothetical HIP projection on the skin, identified by the model over T3 while the 2.5 × 2.5 cm cathodal electrode was placed over O1.

Pads and electrodes were roughly of the same size. In line with current safety guidelines for tDCS applications (Antal et al., 2017; Woods et al., 2016), stimulation parameters were selected to minimize any risk. Specifically, we used a 1 mA current for 20 min with electrodes measuring 2.5 × 2.5 cm (6.25 cm^2^), resulting in a charge density of approximately 192 C/m^2^. This value remains well within acceptable safety thresholds reported in the literature (Antal et al., 2017; Woods et al., 2016). The sham condition consisted of 30 s of active stimulation followed by 19.5 min of sham stimulation.

### 2.5 Procedure

Each participant completed three separate sessions, each corresponding to one of the three stimulation types in a counterbalanced order determined by a Latin square. Sessions were scheduled 1 week apart to minimize potential carryover effects, and all were completed within a maximum period of 3 weeks. At the beginning of the first session, provided written informed consent to take part to the study. Then they completed an interview to verify eligibility for tDCS stimulation. The following description was the same for all the experimental session. Participants seated in a dedicated room, and tDCS electrodes were positioned according to procedure descripted above. Following electrode placement, participants viewed a panoramic (360°) video of the virtual room [the same used by Sulpizio et al. (2013)], which included only the stable objects and omitted the arbitrary objects and the plant. Participants were allowed to watch the video as many times as necessary in order to subsequently draw the room’s layout on paper, indicating the location of the stable elements on the walls. This familiarization procedure was repeated until the participant accurately represented the room’s configuration. Afterward, instructions for the spatial memory task were provided along with example trials for each reference frame condition, ensuring participants understood the task structure. Upon completion of this training phase, tDCS stimulation was initiated. After approximately 5 min of offline stimulation, participants started the task and were left alone in the room after reading the instruction to minimize potential interference or bias from the experimenter. The task was programmed in Matlab (R2021a; MathWorks Inc., Natick, MA, USA).

### 2.6 Data analysis

Based on previous evidence (e.g., Sulpizio et al., 2013), which demonstrated that performance is generally higher at 0° compared to rotated viewpoints (45° and 135°), two separate analyses were conducted on the accuracy, distinguishing between shifting trials (45° and 135°) and no-shift (0°) trials. For both analysis, accuracy was computed as a proportion score, calculated as the number of correct responses for each reference frame divided by the total number of trials in that perspective shift category. Stimulation site (left RSC, left HIP, or sham tDCS), spatial reference frame (viewer-, object-, and room-centered), and, shifting trials, perspective shift (45° and 135°) were considered as independent variables. The Shapiro–Wilk test revealed that accuracy distributions deviated significantly from normality in both no-shift (0°) trials (*W* = 0.253–0.706, *p* < 0.001) and shifting trials (45°, 135°; *W* = 0.352–0.800, *p* < 0.002). Moreover, before performing statistical analysis, we also examined the skewness and kurtosis of the data. Many parameters were greater than −1 and 1, indicating that the data are not normally distributed. A ceiling effect was revealed in some conditions (particularly 0° and viewer-centered trials). This reduced variability and skewed the distributions toward the upper bound, contributing to the observed violations of normality. For this reason and given the violation of normality assumptions, non-parametric analyses were performed. Specifically, Friedman’s ANOVA was used to assess the main effects and interactions, followed by Wilcoxon signed-rank tests for *post hoc* pairwise comparisons. To better appreciate the magnitude of the observed effects, we included the effect size (r) for key comparisons. All these previous analyses was conducted with SPSS (Statistical Package for Social Sciences; IBM).

## 3 Results

A Friedman test was conducted to compare accuracy across the three stimulation conditions (RSC, HIP, and sham). Results indicated no significant differences between conditions [*χ*^2^(2) = 1.65, *p* = 0.44], suggesting that stimulation type did not globally affect performance.

### 3.1 No-shift (0°) trials

**Figure 2:**
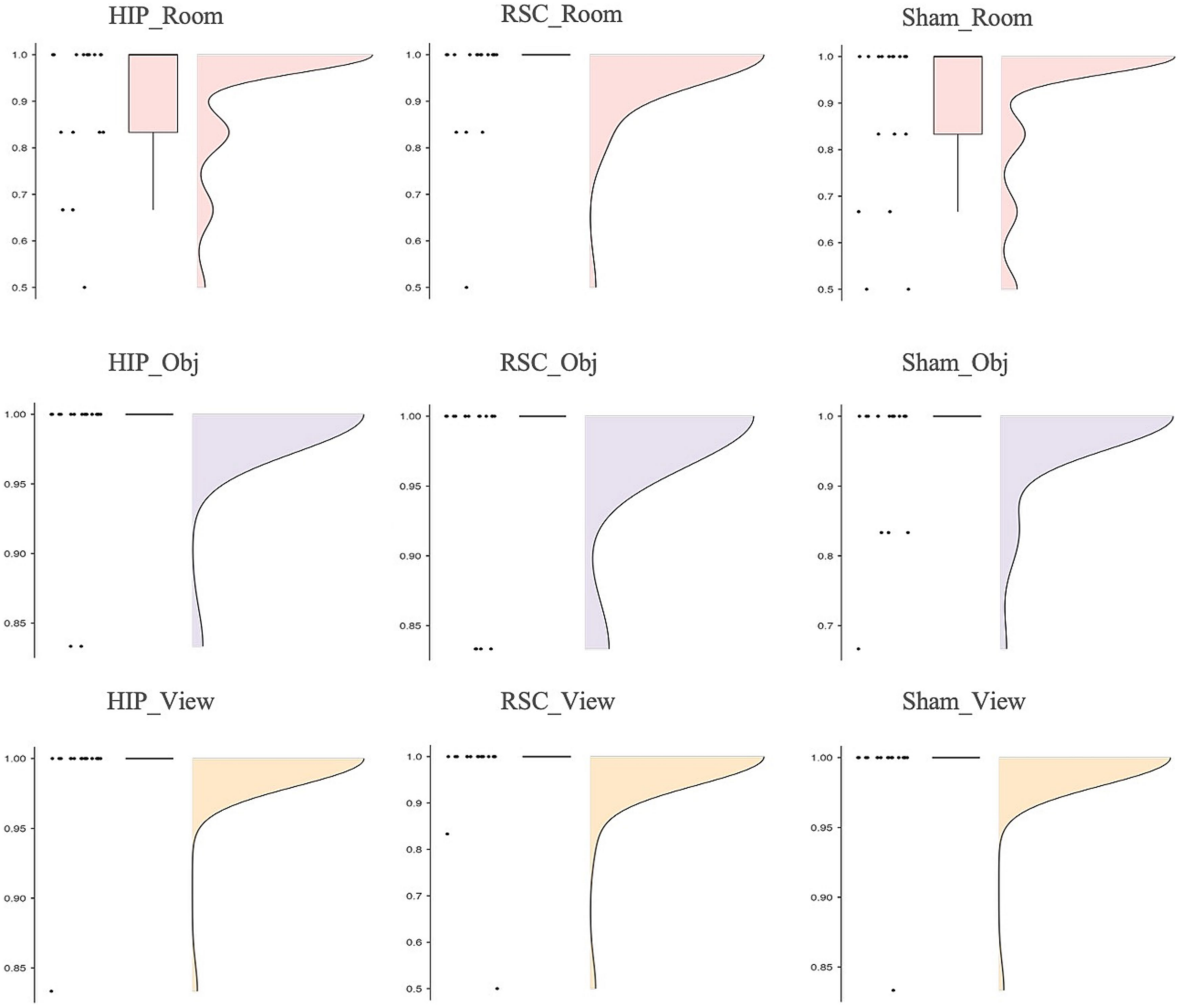
Accuracy as function of stimulation sites (HIP, RSC, sham) separately for room (top row), object (middle row) and viewer (bottom row) in no-changing trials (0°).

Friedman’s analysis of variance revealed a significant effect of stimulation type and spatial reference frame on accuracy [*χ*^2^(8) = 17.911, *p* = 0.022; Figure 2]. Post-hoc Wilcoxon signed-rank tests showed that, under hippocampal stimulation, accuracy was significantly higher in the object-centered block compared to the room-centered block (*Z* = −2.124, *p* = 0.034, *r* = 0.50), and in the viewer-centered block compared to the room-centered block (*Z* = −2.157, *p* = 0.031, *r* = 0.51). Under sham stimulation, accuracy was significantly higher in the viewer-centered block compared to the room-centered block (*Z* = −2.264, *p* = 0.024, *r* = 0.53). All other comparisons did not reach statistical significance. All the statistics parameters are reported in Table 1.

**Table 1:** Mean accuracy (±SD) and median (IQR) as function of stimulation conditions (HIP, RSC, sham) and reference frame (room, object, and viewer) under 0° rotation (no-shift trials).

Reference frame	HIP		RSC		Sham	
	M (SD)	MEDIAN (IQR)	M (SD)	MEDIAN (IQR)	M (SD)	MEDIAN (IQR)
Room	0.89 (0.15)	1.0 (0.16)	0.94 (0.12)	1.0 (0.00)	0.87 (0.17)	1.0 (0.16)
Object	0.98 (0.05)	1.0 (0.00)	0.96 (0.07)	1.0 (0.00)	0.95 (0.09)	1.0 (0.00)
Viewer	0.99 (0.03)	1.0 (0.00)	0.99 (0.03)	1.0 (0.00)	0.96 (0.12)	1.0 (0.00)

### 3.2 Shifting trials (45°, 135°)

**Figure 3:**
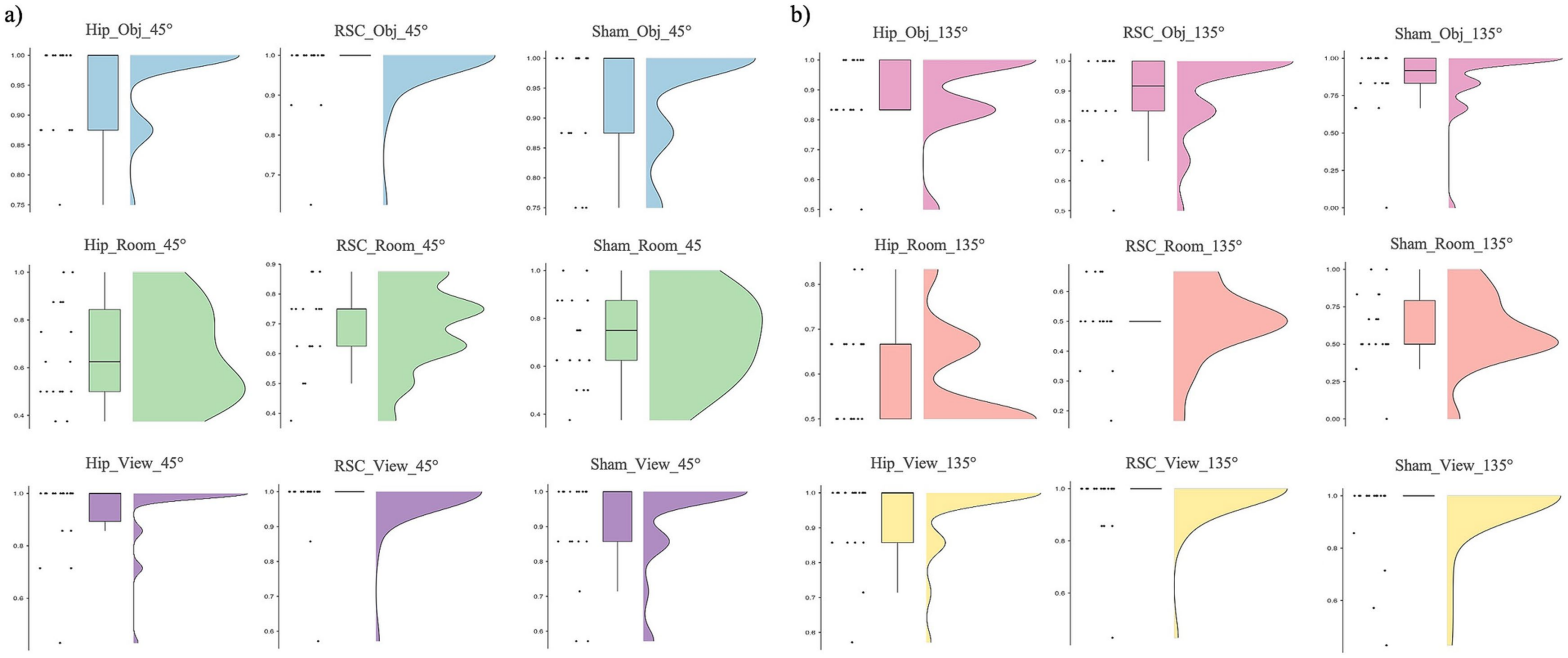
Accuracy as function of stimulation sites (HIP, RSC, sham), separately for room (top row), object (middle row) and viewer (bottom row) in changing trials: **(a)** 45° and **(b)** 135°.

Friedman’s test revealed a significant effect of stimulation type, spatial reference frame, and viewpoint rotation on accuracy [*χ*^2^(17) = 161.878, *p* < 0.001; Figure 3]. Overall, accuracy was highest in the object- and viewer-centered blocks and lowest in the room-centered block, especially when the perspective shifted by 135°.

Post-hoc Wilcoxon signed-rank tests showed that during HIP stimulation, participants were more accurate in the object-centered block at 45° compared to 135° (*Z* = −2.453, *p* = 0.014, *r* = 0.58). A similar sensitivity to rotation was found under RSC stimulation: accuracy in the room-centered block was higher at 45° than at 135° (*Z* = −3.113, *p* = 0.002, *r* = 0.73), and object-centered performance was likewise higher at 45° than at 135° (*Z* = −2.556, *p* = 0.011, *r* = 0.60). No other within-condition differences reached significance. A comparison across stimulation conditions revealed that, in the room-centered block at 135°, accuracy was significantly lower under RSC stimulation compared to both HIP (*Z* = −2.377, *p* = 0.017, *r* = 0.56) and sham stimulation (Z = −2.111, *p* = 0.035, *r* = 0.50). Finally, the systematic disadvantage of the room-centered frame emerged when comparing across reference frames. At 45°, accuracy was significantly lower in room-centered block than in both the viewer- and object-centered blocks. This pattern was observed across all stimulation conditions: RSC stimulation (viewer vs. room: *Z* = −3.702, *p* < 0.001, *r* = 0.87; object vs. room: *Z* = −3.649, *p* < 0.001, *r* = 0.86), HIP stimulation (object vs. room: *Z* = −3.405, *p* = 0.001, *r* = 0.80; viewer vs. room: *Z* = −3.367, *p* = 0.001, *r* = 0.79), and sham stimulation (viewer vs. room: *Z* = −3.212, *p* = 0.00, *r* = 0.76; object vs. room: *Z* = −3.156, *p* = 0.002, *r* = 0.74).

At 135°, the same disadvantage for room-centered judgments was observed: under RSC stimulation, accuracy was significantly lower in the room-centered block than both the viewer-centered (*Z* = −3.736, *p* < 0.001, *r* = 0.88) and object-centered blocks (*Z* = −3.841, *p* < 0.001, *r* = 0.91). Comparable results were obtained under HIP stimulation (viewer vs. room: Z = −3.743, *p* < 0.001, *r* = 0.88; object vs. room: *Z* = −3.525, *p* < 0.001, *r* = 0.83) and sham stimulation (viewer vs. room: *Z* = −3.464, *p* = 0.001, *r* = 0.82). All the statistics parameters are reported in Table 2.

**Table 2:** Mean accuracy (±SD) and median (IQR) as function of stimulation conditions (HIP, RSC, sham) and reference frame (room, object, and viewer) for the shifting-trials (45° and 135°).

Reference frame	HIP				RSC				Sham			
	45°		135°		45°		135°		45°		135°	
	M (SD)	Mdn (IQR)	M (SD)	Mdn (IQR)	M (SD)	Mdn (IQR)	M (SD)	Mdn (IQR)	M (SD)	Mdn (IQR)	M (SD)	Mdn (IQR)
Room	0.66 (0.20)	0.63 (0.34)	0.61 (0.11)	0.67 (0.17)	0.69 (0.14)	0.67 (0.17)	0.50 (0.13)	0.50 (0.17)	0.72 (0.18)	0.67 (0.17)	0.60 (0.24)	0.67 (0.25)
Object	0.95 (0.08)	1.00 (0.13)	0.86 (0.15)	0.83 (0.17)	0.97 (0.09)	1.00 (0.13)	0.88 (0.15)	0.83 (0.25)	0.92 (0.10)	1.00 (0.13)	0.84 (0.25)	0.83 (0.25)
Viewer	0.92 (0.16)	1.00 (0.11)	0.93 (0.12)	1.00 (0.14)	0.97 (0.10)	1.00 (0.11)	0.94 (0.14)	1.00 (0.17)	0.90 (0.15)	1.00 (0.11)	0.92 (0.17)	1.00 (0.14)

## 4 Discussion

The aim of this study was to assess the contribution of RSC and HIP in spatial memory task. The results demonstrated that stimulation RSC modulates participants’ spatial memory performance in a condition-specific manner. Specifically, during RSC stimulation, participants showed significantly reduced accuracy in the room-centered block with a viewpoint shift of 135°, both relative to sham and HIP stimulation. This result is consistent with previous neuroimaging study employing the same paradigm, which revealed selective activation of the RSC during stable environmental frame (Sulpizio et al., 2013). Similar results supporting representations tied to stable environmental features were also obtained with other paradigm (Galati et al., 2010) as well as lesion studies showing that RSC damage compromises spatial reorientation and topographical memory (Song et al., 2020; Valenstein et al., 1987). Therefore, our results extend previous correlational evidence from neuroimaging and lesion studies by offering a causal demonstration of the role of RSC in critical viewpoint-dependent spatial updating anchored to stable landmarks. Moreover, our results suggest that the RSC is reliably engaged when spatial judgments depend on stable environmental cues. However, the drop in performance under RSC stimulation when stimuli were presented with a viewpoint shift of 135° appears to contradict such literature, which has described the RSC as critical for coding object positions relative to stable landmarks and for retrieving these positions after perspective changes (Chrastil et al., 2018; Sulpizio et al., 2013).

A plausible interpretation is that the RSC was functionally engaged in these trials and that stimulation interfered with its normal contribution to offline spatial updating, a process theorized to require transformation of egocentric representations into allocentric coordinates and the integration of partial environmental cues into a coherent spatial model (Byrne et al., 2007; Vann et al., 2009). Galati et al. (2010) suggested that the RSC is particularly active when the environment must be reconstructed mentally in the absence of complete sensory input, precisely the case of the viewpoint shift of 135°, where snapshots include only partial room features. In this view, anodal stimulation may have disrupted the functional interactions between the RSC and connected regions such as the HIP and the posterior parietal cortex by introducing neural noise or unbalancing network-level connectivity. Brunyé (2018) pointed out that NIBS can modulate a wide range of spatial processes, including mental rotation, visualization, and navigation, but that such effects are strongly contingent on the functional connectivity of the stimulated area with deeper nodes such as the RSC and HIP. In detail, given that the RSC serves as a hub for integrating egocentric and allocentric information (Byrne et al., 2007; Ekstrom et al., 2014), perturbing its excitability could have interfered with network dynamics, thereby reducing rather than enhancing efficiency (Chen et al., 2024).

Notably, this condition was the only one in which a significant stimulation-related modulation was observed, underscoring the specificity of the RSC’s role in viewpoint-dependent updating anchored to environmental landmarks. By contrast, stimulation of the HIP failed to modulate spatial memory performance. Although the HIP plays a well-established role in the encoding and retrieval of allocentric spatial representations (Bird and Burgess, 2008; Burgess et al., 2002), its contribution may be less relevant in the specific demands of the present task, which emphasized rapid offline updating of spatial scenes from novel viewpoints. Our results may be also explained considering the functional specialization of the two hemispheres: although the left hippocampus contribute to spatial memory, particularly when spatial information is embedded within narratives or route-based sequences, the right hippocampus is more strongly implicated in this function (Burgess et al., 2002; Epstein et al., 2017; Maguire and Frith, 2003). Moreover, such processes may have been only marginally engaged by the current paradigm, which requires rapid spatial updating and relational mapping.

With respect to the reference frame, more broadly, results from no-shift trials revealed that participants were more accurate in the viewer-centered than the room-centered block under sham stimulation, consistent with the idea that egocentric representations, anchored directly to the observer, require less transformation and are cognitively less demanding (Burgess et al., 2006). A similar advantage for viewer-centered over room-centered judgments was found under HIP stimulation but not under RSC stimulation, possibly indicating that RSC engagement may have reduced the performance gap between allocentric and egocentric frames. In support of this, the RSC has been proposed as a mediator between parietal egocentric and medial temporal allocentric systems (Byrne et al., 2007; Ekstrom et al., 2014), and its involvement might attenuate the typical dominance of egocentric processing. Accuracy was also significantly higher in object-based than room-based frames under hippocampal stimulation, in line with the role of HIP in constructing spatial representations that integrate identity (“what”) and location (“where”) information (Bird and Burgess, 2008; Mao et al., 2017).

Trials involving perspective changes (45° and 135°) were inherently more complex, especially in object- and room-centered block, requiring participants to mentally reconstruct the spatial layout, retrieve the allocentric representation, and apply a mental self-rotation to align viewpoints, a process that taxes spatial working memory and visual imagery (Chrastil et al., 2018). In all three stimulation conditions and across both rotation angles, viewer-based performance was superior to room-based, reflecting the stability of egocentric relations across perspective shifts. Moreover, accuracy was consistently lower at 135° compared to 45°, likely due to the increased cognitive load and initial disorientation caused by the larger viewpoint transformation (Alexander and Nitz, 2017; Sulpizio et al., 2013). Taken together, the results provide causal evidence that the RSC plays a selective and critical role in spatial memory updating, when tasks require, target positions anchored to stable environmental cues from a novel perspective. This supports and extends prior correlational findings and introduces new questions about how non-invasive stimulation might differentially modulate deep brain systems depending on cognitive context. Although the direction of the stimulation effect (i.e., reduced performance under anodal stimulation) may seem counterintuitive, it reflects growing recognition that tDCS effects are not uniformly excitatory or facilitative (Jacobson et al., 2012), especially when targeting integrative hub regions like the RSC. Due to its deep anatomical location, the effects of stimulation over the RSC are still not well understood and require further exploration. A meta-analysis examining the polarity effects of tDCS across motor and cognitive domains (Jacobson et al., 2012) found that the classic anodal-excitation/cathodal-inhibition (AeCi) effect is significantly weaker in cognitive tasks. Furthermore, Purpura and McMurtry (1965) reported that in deeper cortical layers, anodal stimulation may lead to neuronal inhibition, while cathodal stimulation may induce excitation. Supporting this complexity, other studies have shown that the behavioral effects of tDCS may be influenced by task difficulty and individual cognitive abilities, with anodal stimulation occasionally resulting in performance impairments (Jones and Berryhill, 2012). In line with this, the present study found that trials requiring room-centered encoding under perspective change were particularly challenging, raising the possibility that stimulation effects were modulated by task complexity.

### 4.1 Limits and future directions

The retrosplenial cortex (RSC) remains a relatively underexplored brain region, and to date, to our knowledge, no prior study has employed non-invasive brain stimulation to investigate its functions in humans. It remains unclear which cognitive mechanisms were affected by tDCS in this context, and how they interacted with stimulation of the RSC. Future studies may be useful to understand the effect of the right RSC stimulation, given its stronger involvement in spatial updating and environmental representation as suggested by prior neuroimaging studies (Galati et al., 2000; Sulpizio et al., 2013). In particular, Sulpizio and colleagues proposed that offline updating of a stable environmental representation may be more strongly supported by the right hemisphere RSC. Moreover, no significant behavioral effects were observed following HIP stimulation, despite the well-established role of this structure in spatial and episodic memory processes. This null result warrants further investigation, as it may reflect technical challenges in targeting the HIP with tDCS (e.g., field strength at depth, variability across participants). Indeed, both stable and variable inter-individual differences (morphological and genetic features, participants’ engagement/baseline capacity) could determine non-linear and state-dependent tDCS effects that could partially account for the heterogeneity of our results (Vergallito et al., 2022). Moreover, functional differences between the left and right HIP could also play a role in determining null effects. As reported above, stimulation of the right HIP could be explored, as it has been more consistently implicated in the encoding and retrieval of object positions and spatial relationships within large-scale environments (Burgess et al., 2002). Thus, future research should consider stimulating both the right and left HIP to directly compare their respective contributions to spatial memory and to better understand the functional lateralization of this region.

Additional methodological limitations should also be considered. The spatial memory task used in this study involved a substantial asymmetry in difficulty: trials with no perspective shift (0°) were considerably easier than those involving viewpoint changes (45°, 135°), necessitating separate analyses. Furthermore, accuracy also varied across spatial reference frames, suggesting a caution in the interpretation of results. The task appears to be either too easy with an accuracy higher than 90% for no-shift trials or too difficult in shifting trials with room reference. This pattern replicated the original study (Sulpizio et al., 2013), but it also resulted in ceiling effects in some conditions (particularly in no-shift trials) and near-chance performance in others, which may have reduced statistical sensitivity and limited the informativeness of certain comparisons. This variability may have influenced the observed stimulation effects and their interpretation.

Another limitation pertains to the sample size. In the present study, statistical power was estimated for a within subject repeated-measures ANOVA model. As the data violated the assumptions of normality, non-parametric tests were used. Consequently, the power analysis was no longer adequate. For these reasons, it is essential that our findings are interpreted with the utmost caution.

Furthermore, we calculated statistical power by planning to balance gender. This did not allow us to verify any differences between males and females. Since gender differences have been found in spatial memory (Yuan et al., 2019), future studies could assessed gender differences with larger sample.

Finally, although our parameters complied with current safety recommendations, it should be noted that scalp-based tDCS inevitably produces diffuse current spread, thereby limiting anatomical specificity. This limitation is particularly relevant when attempting to modulate deep brain regions such as the HIP and RSC, where stimulation effects may partly reflect indirect network-level influences rather than focal modulation. Stimulation parameters used in the present study followed the same approach already used in literature (see, e.g., Sagliano et al., 2019) to stimulate the insular cortex and reporting an effect of the anodal stimulation in interfering with interoceptive process. Considering these challenges, future studies could benefit from the application of alternative NIBS techniques. In particular, high-definition tDCS may provide enhanced spatial precision, while transcranial magnetic stimulation (TMS) could allow time-locked interference with functional processing within hippocampal and retrosplenial circuits. Moreover, high-definition tDCS could be used in future studies to assess the involvement of other cortical regions, such as the medial temporal cortex and adjacent antero-medial occipital lobe (fusiform, lingual and posterior parahippocampal gyrus), and the precuneus also involved in spatial judgement (Committeri et al., 2004; Sulpizio et al., 2013).

## 5 Conclusion

In conclusion, the present study represent the first attempt to provide causal evidence of the involvement of the RSC cortex in spatial memory. Here we provided a causal demonstration of the role of RSC in critical viewpoint-dependent spatial updating anchored to stable landmarks.

Anodal tDCS over the left RSC altered performance on a spatial localization task, particularly in conditions requiring viewpoint changes, suggesting its role in integrating multiple perspectives within a coherent spatial framework. The RSC appears to support spatial orientation and the transformation between egocentric and allocentric coordinates, especially when environmental cues are stable across views (Galati et al., 2010; Sulpizio et al., 2013, 2016; Byrne et al., 2007). Nevertheless, the precise mechanisms by which anodal tDCS affects this region remain to be clarified. Finally, the absence of observable effects under hippocampal stimulation underscores the need for further research to understand the neuromodulator potential of tDCS on this structure.

## Data Availability

Publicly available datasets were analyzed in this study. Queries regarding the datasets should be directed to the corresponding author.
