# Supplementary material for: The role of the hippocampus and retrosplenial cortex in spatial memory: a double blind anodal transcranial direct current stimulation study
Source: Front Hum Neurosci. 2025 Oct 20;19:1661310. doi: 10.3389/fnhum.2025.1661310 (PMC12580218; doi:10.3389/fnhum.2025.1661310)
Supplement: Supplementary file 1 [file Data_Sheet_1.docx]

**Supplementary Materials**

*Computational model of the electric field amplitude distribution*

The electric field (**E**) distribution was obtained by computational method applied to a realistic human model. Specifically, simulations were conducted using the simulation platform Sim4Life simulation platform (developed by SPEAG, Schmid & Partner Engineering AG, Zurich, Switzerland; www.speag.com), solving the Laplace equation to determine the electric potential (φ$\phi$) distribution inside the human tissues (1)

1.

where $\sigma$σ is the electrical conductivity of the human tissues. The $\vec{E}$**E** amplitude distributions were obtained by means of the following relation (2):

1.

A multimodal imaging-based detailed anatomical model (Iacono et al., 2015), of the human head and neck, named “MIDA” was imported in the simulation platform Sim4Life. The model was segmented and reconstructed at an isotropic resolution equal to 0.5 mm, which allowed to distinguish the tissues mainly involved in the stimulation protocols, such as the two hippocampi. Since the retrosplenial cortex, which is one of the main targets of the stimulation, was not segmented in the original MIDA model, it was identified by Montreal Neurological Institute coordinates. The dielectric properties of each tissue were assigned based on data at low frequency, following an approach already used in literature (Parazzini et al., 2017).

The electrodes were modeled as rectangular pads conductors (σ =5.9x10^7^ S/m) placed above a rectangular sponge (σ = 1.4 S/m) of the same dimensions. The potential difference between the electrodes was adjusted to inject a total current of 1 mA.

Nine different electrode montages were compared with the aim to induce the higher and the more widespread **E** amplitude distribution over the RSC area. With reference to the 10-20 EEG International System, the electrodes were simulated as:

1. 2.5 × 2.5 cm^2^ active electrode centered on the middle point between T3 and T5 and reference electrode (5 × 5 cm^2^) centered on Fp1
2. 2.5 × 2.5 cm^2^ active electrode centered on the RSC projection on the model skin and reference electrode (5 × 5 cm^2^) centered on Fp1
3. 5 × 5 cm^2^ active electrode centered on the middle point between T3 and T5 and reference electrode (5 × 5 cm^2^) centered on Fp1
4. 5 × 5 cm^2^ active electrode centered on the RSC projection on the model skin and reference electrode (5 × 5 cm^2^) centered on Fp1
5. 2.5 × 2.5 cm^2^ active electrode centered on the middle point between T3 and T5 and reference electrode (2.5 × 2.5 cm^2^) centered on Fp1
6. 2.5 × 2.5 cm^2^ active electrode centered on the RSC projection on the model skin and reference electrode (2.5 × 2.5 cm^2^) centered on Fp1
7. 2.5 × 2.5 cm^2^ active electrode centered on the hippocampus projection on the model skin and reference electrode (2.5 × 2.5 cm^2^) centered on Fp1
8. 2.5 × 2.5 cm^2^ active electrode centered on the RSC projection on the model skin and reference electrode (2.5 × 2.5 cm^2^) centered on O1
9. 2.5 × 2.5 cm^2^ active electrode centered on the hippocampus projection on the model skin and reference electrode (2.5 × 2.5 cm^2^) centered on O1

| A 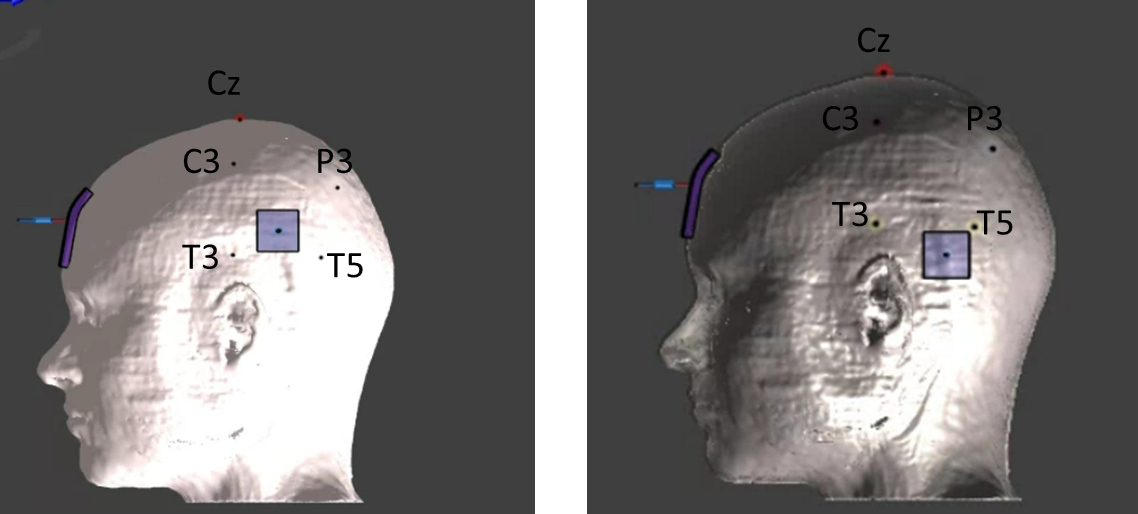 | B 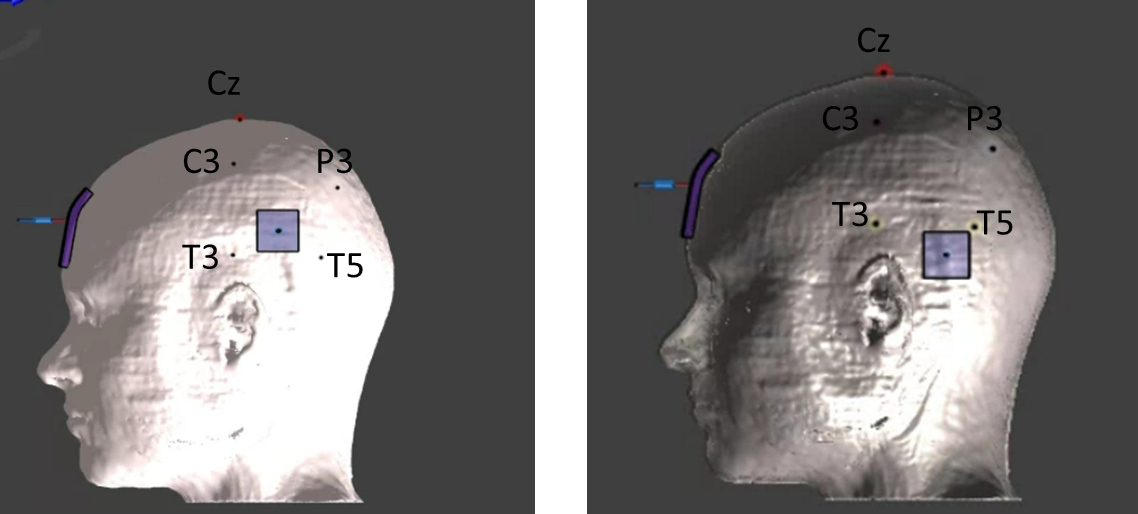 | C 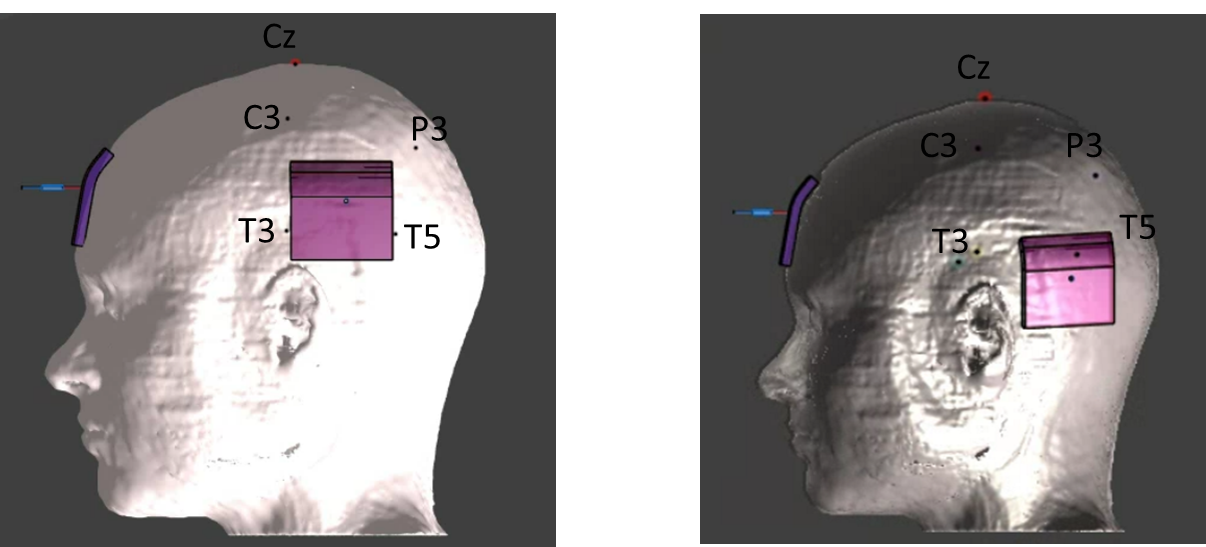 |
| --- | --- | --- |
| D 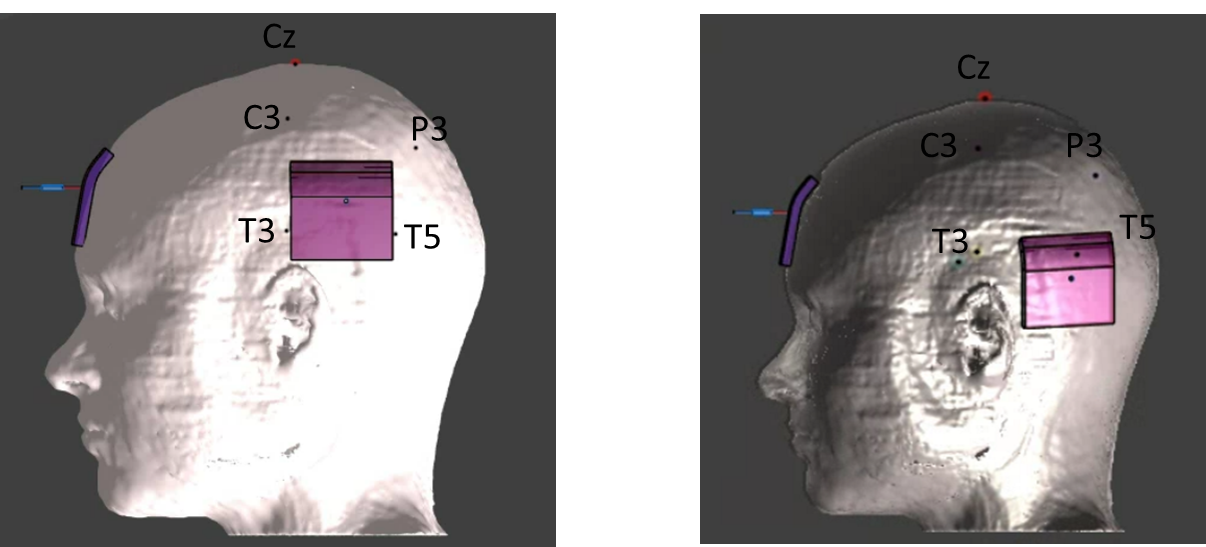 | E 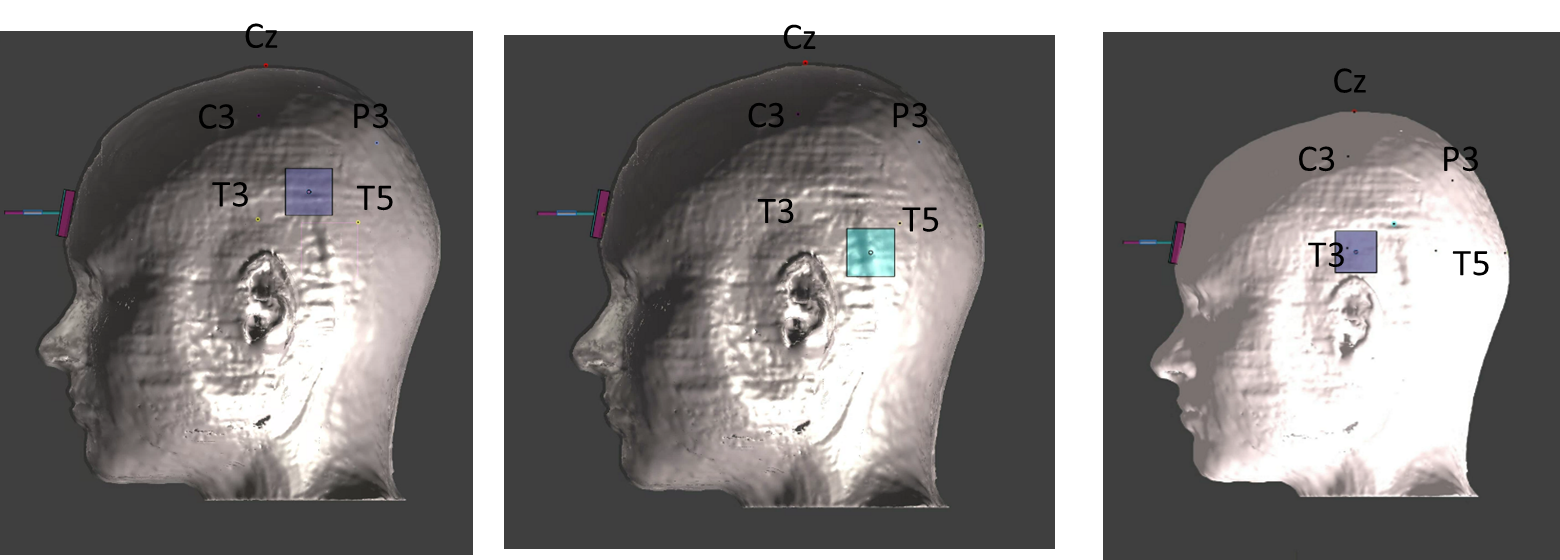 | F 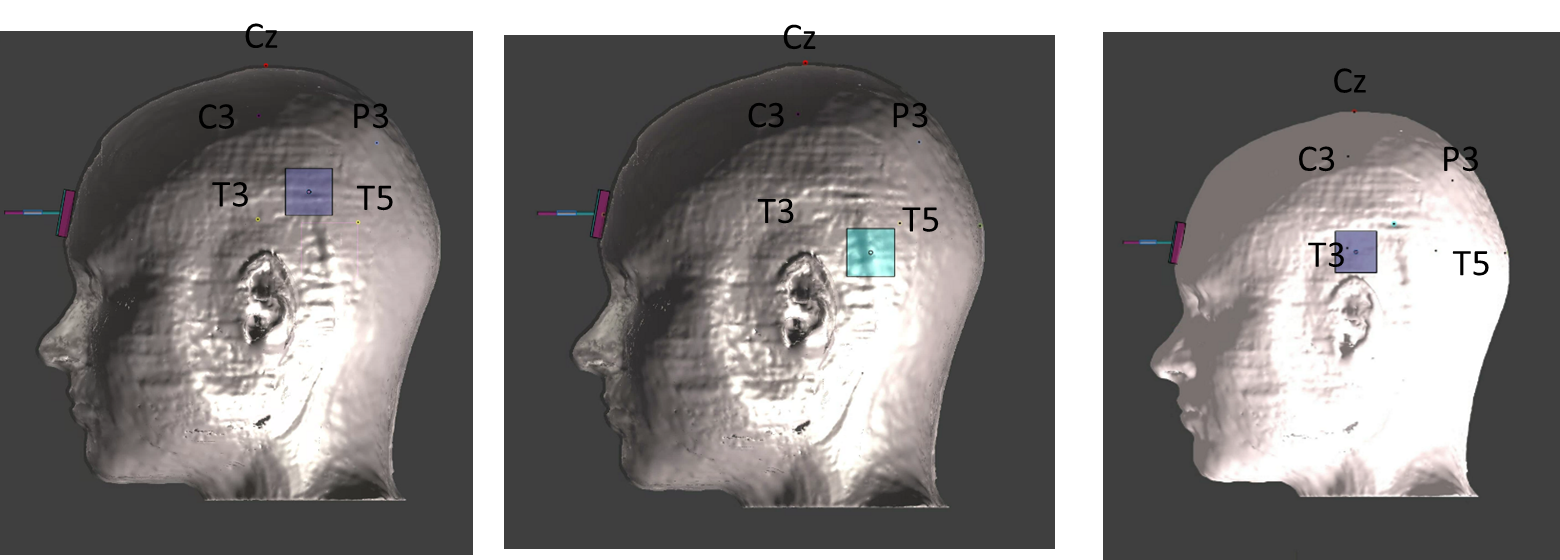 |
| G 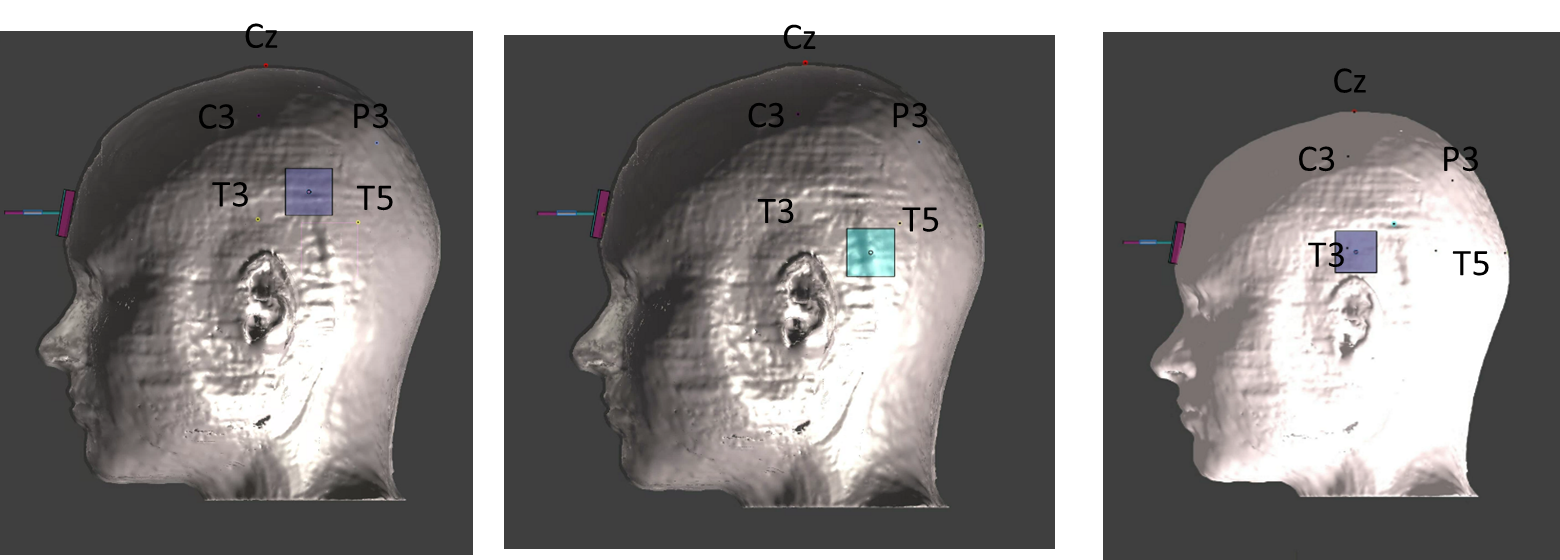 | H 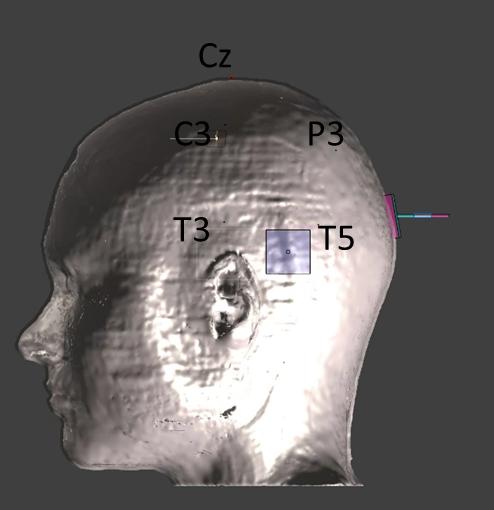 | I 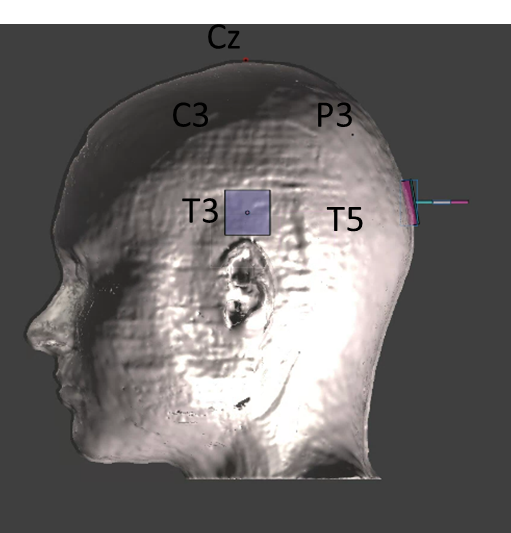 |

Fig 1. The nine simulated electrode montages.

The comparison between the nine electrode montages was performed quantifying the **E** amplitude induced by each electrode montage in the RSC and hippocampus (i.e. our targets) and in other three tissues, i.e. brain white matter, brain gray matter and cerebellum. In particular, the following parameters were compared:

- the 50^th^ and the 99^th^ percentile of the **E** amplitude distribution induced in each tissue;
- Percentage of each tissue volume where the E amplitude is higher than the 50% (V50) or 70% (V70) of the 99^th^ of the E amplitude.

Fig. 2 and 3 show the percentile of the **E** amplitude distribution and the percentage V50 and V70 induced over the different tissues for each electrodes montage.

| 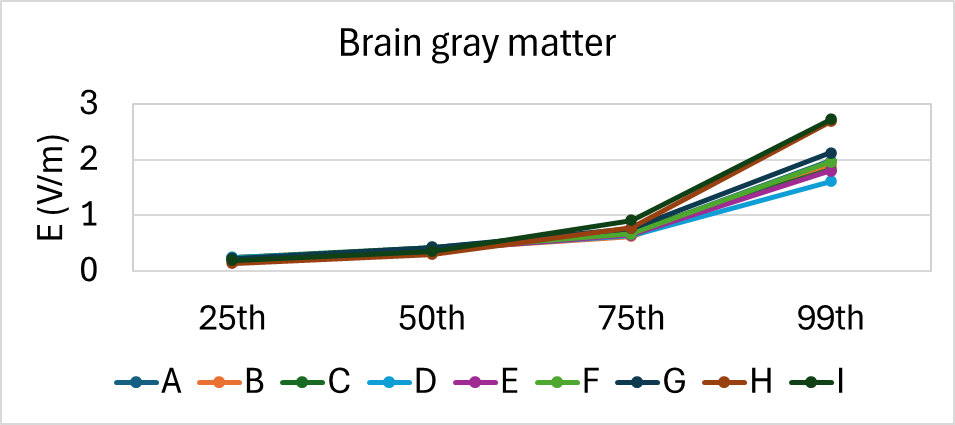 | 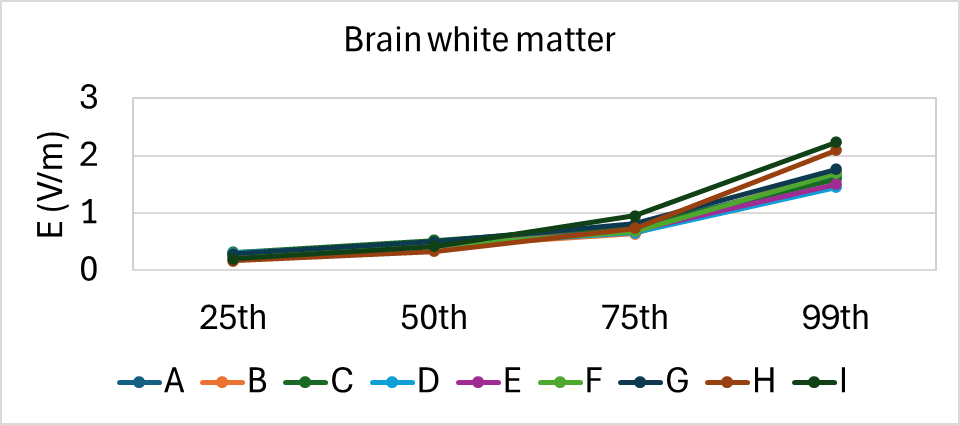 |
| --- | --- |
| 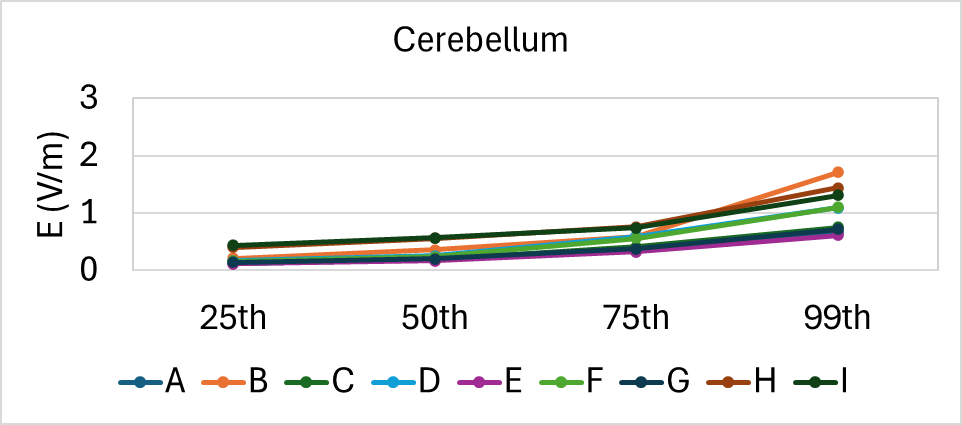 | 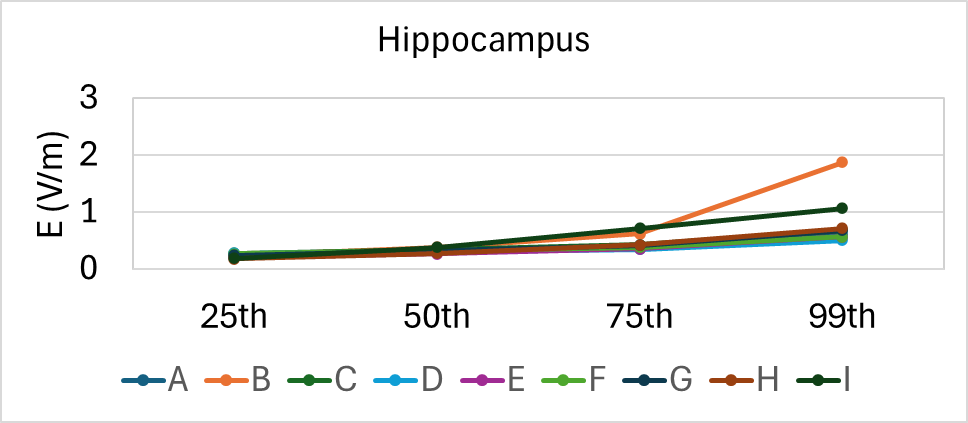 |
| 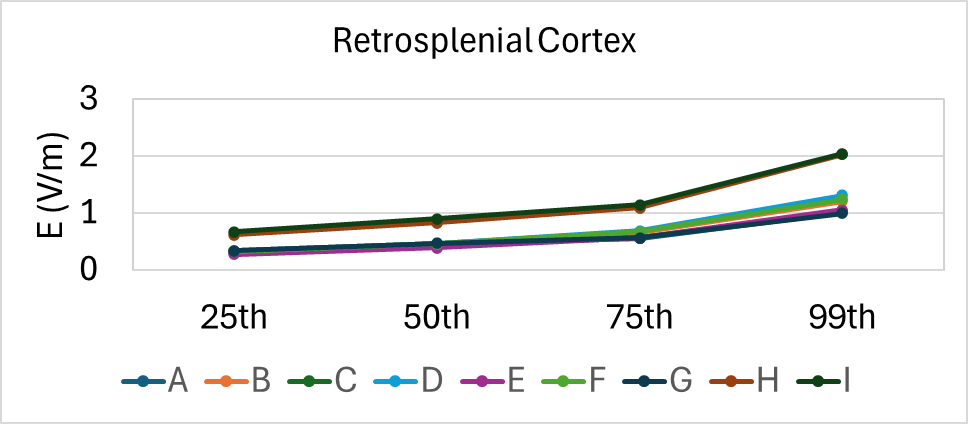 | |

Fig 2. E field distributions: descriptive statistics.

| 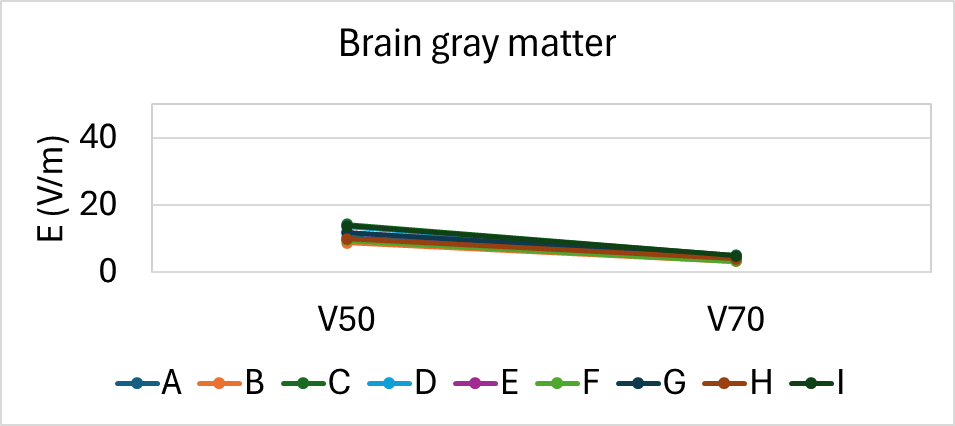 | 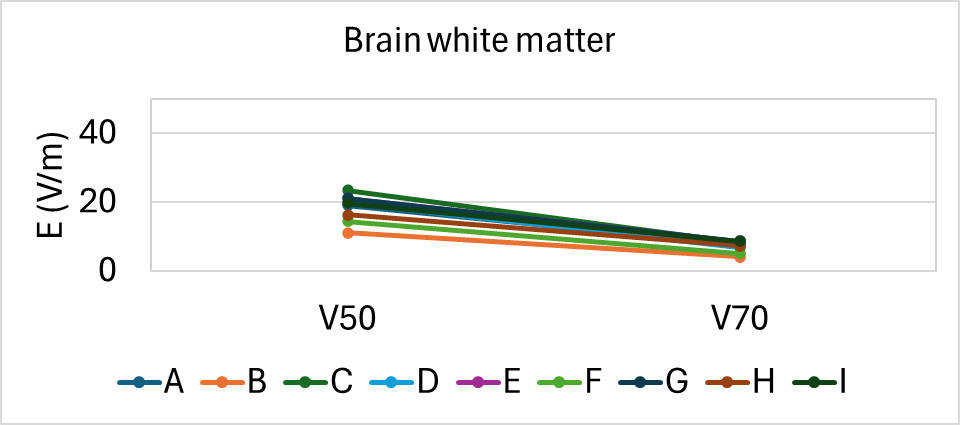 |
| --- | --- |
| 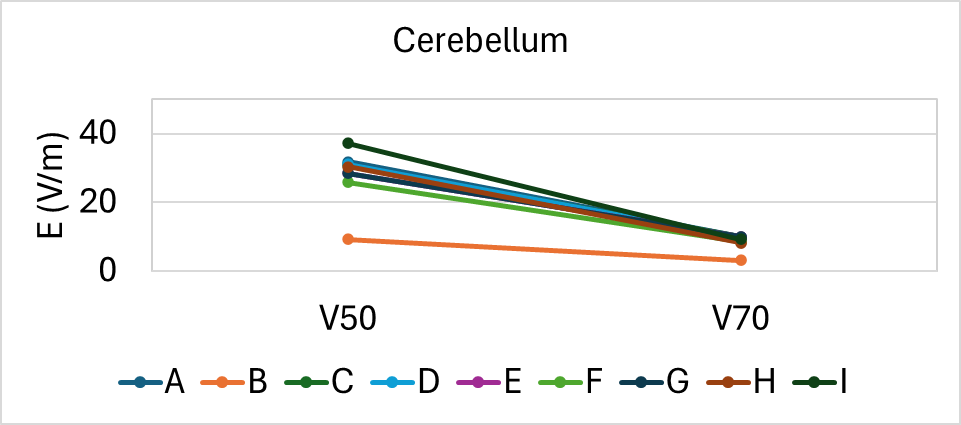 | 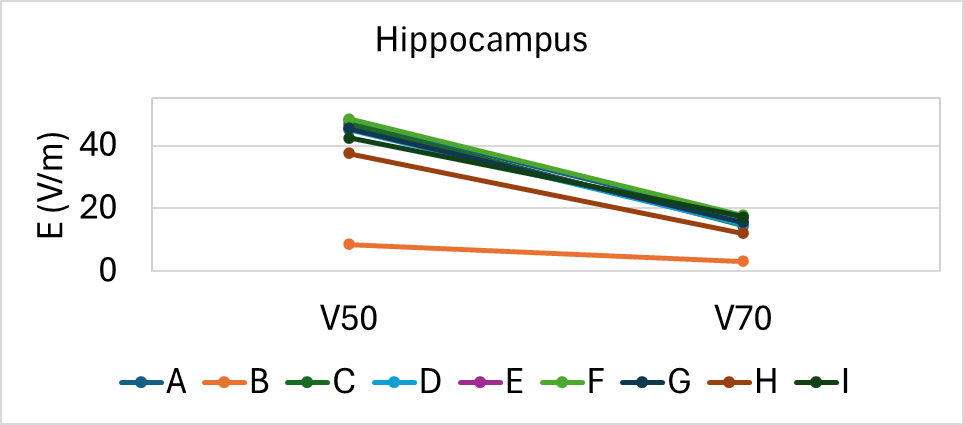 |
| 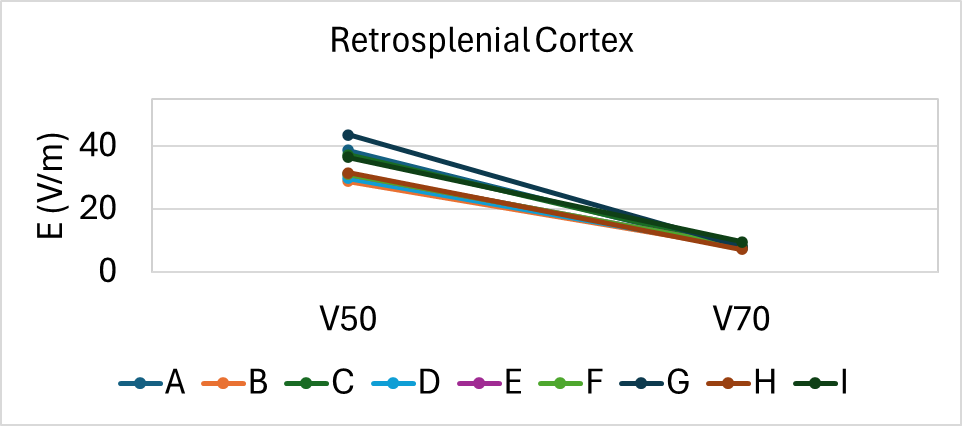 | |

Fig 3. Percentage of each tissue volume where the E amplitude is higher than the 50% (V50) or 70% (V70) of the 99^th^ of the E amplitude.

Data showed that smaller electrodes allowed reaching a higher amplitude of E distributions inside the tissues of interest. In particular, electrodes montages H and I were identified as the most promising for targeting RSC and hippocampus, respectively.

ACKNOWLEDGMENTS

The authors wish to thank Schmid and Partner Engineering AG (www.speag.com) for having provided the simulation softwares X/SIM4Life.

**References**

Iacono, M.I., et al (2015). MIDA: A Multimodal Imaging-Based Detailed Anatomical Model of the Human Head and Neck. PLoS ONE 10(4): e0124126. doi:10.1371/journal.pone.0124126

Parazzini M, Fiocchi S, Cancelli A, Cottone C, Liorni I, Ravazzani P, Tecchio F. (2017) A Computational Model of the Electric Field Distribution due to Regional Personalized or Non-Personalized Electrodes to Select
